# An Obscure Presence of Gastroduodenal Involvement in a Newly Diagnosed Ileocolonic Crohn's Disease Patient

**DOI:** 10.1155/2022/2200438

**Published:** 2022-12-27

**Authors:** Clive Jude Miranda, Murad Hayatt Ali, Muddasir Ayaz, Yousef Soofi, Thomas Christopher Mahl

**Affiliations:** Department of Gastroenterology, University at Buffalo, 955 Main Street, Buffalo 14203, New York, USA

## Abstract

Whereas typical Crohn's disease is confined to the terminal ileum and presents with abdominal pain and diarrhea, gastroduodenal manifestations of Crohn's disease are rare, with often asymptomatic patient presentations and inconclusive diagnostic testing. It is, however, a more severe form of Crohn's disease and thus warrants treatment with steroids and biologics much earlier than its ileocolonic counterpart. We present the case of a young, otherwise healthy, male with newly diagnosed ileocolonic Crohn's disease with concurrent gastroduodenal involvement that initially failed management with biologic agents. We discuss the clinical manifestations and often obscure pathology of gastroduodenal Crohn's disease and highlight the necessity of performing a concurrent esophagogastroduodenoscopic evaluation on newly diagnosed ileocolonic Crohn's disease to assess the presence of upper gastrointestinal involvement.

## 1. Introduction

Crohn's disease (CD) is an idiopathic, multisystem, inflammatory disease with an etiology intertwined by environmental, microbial, and genetic factors characterized by transmural and segmental involvement of the gastrointestinal (GI) tract, anywhere from the mouth to the anus [1]. Afflicted patients experience episodes of remission and relapse throughout the course of their lives, with the most common complaints including epigastric pain, nausea, vomiting, and weight loss. The disease is most definitively diagnosed with endoscopic evaluation, with aphthous, longitudinal ulcers, and cobblestoning being typical endoscopic findings, and noncaseating granulomas being a hallmark of pathology [2, 3].

Gastroduodenal CD affects 0.5–4% of all CD patients [4], with the majority of such patients having concurrent involvement of the colon with isolated gastroduodenal involvement accounting for less than 0.07% of all CD patients [5, 6]. Pathology is more obscure in the upper GI tract, and noncaseating granulomas are not necessarily needed for confirmatory diagnosis. Though many patients are asymptomatic, gastroduodenal involvement indicates a more severe form of CD and thus warrants steroidal treatment and biological agents earlier in the disease course. Most patients respond well to medical therapy, with surgery indicated for perforation, bleeding, or significant structural disease causing obstructive complications. Here, we present a case of a healthy, young male presenting with new onset gastroduodenal Crohn's disease with concurrent colonic involvement that responded well to biological therapy. We aim to describe the often obscure endoscopic and pathological features associated with gastroduodenal CD and highlight the importance of including upper endoscopy in newly diagnosed ileocolonic CD patients.

## 2. Case Presentation

A 30-year-old male with no prior medical history presented to the Emergency Departmentfor ongoing hematochezia with watery diarrhea, cramping abdominal pain, vomiting, and poor intake for the past 3 months resulting in an unintentional 30 lb weight loss. He worked as a plumber and denied any family history of inflammatory bowel disease. Upon presentation, temperature was elevated at 100.4F with a leukocytosis of 12,000/mm^3^ with neutrophilic predominance. CRP was elevated at 50, stool lactoferrin was positive and calprotectin was 2562. CT angiography showed thickening of the left hemi-colon from mid-transverse to rectosigmoid colon with diffuse reactive adjacent mesenteric lymph nodes, suggestive of acute colitis.

**Figure 1:**
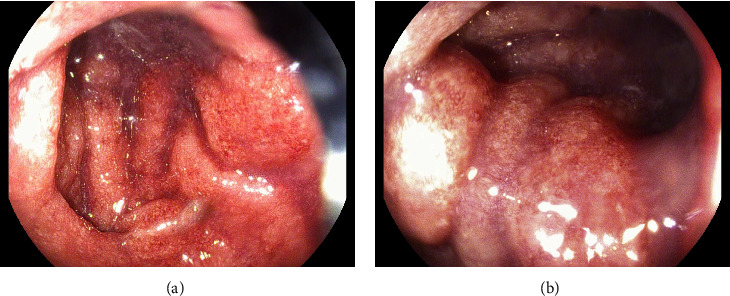
Diffuse ulceration and friability in the rectum and sigmoid colon.

**Figure 2:**
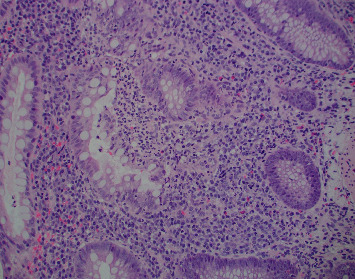
Rectal mucosa with chronic active inflammation with plasma cells and neutrophilic crypts consistent with Crohn's disease, 200x.

**Figure 3:**
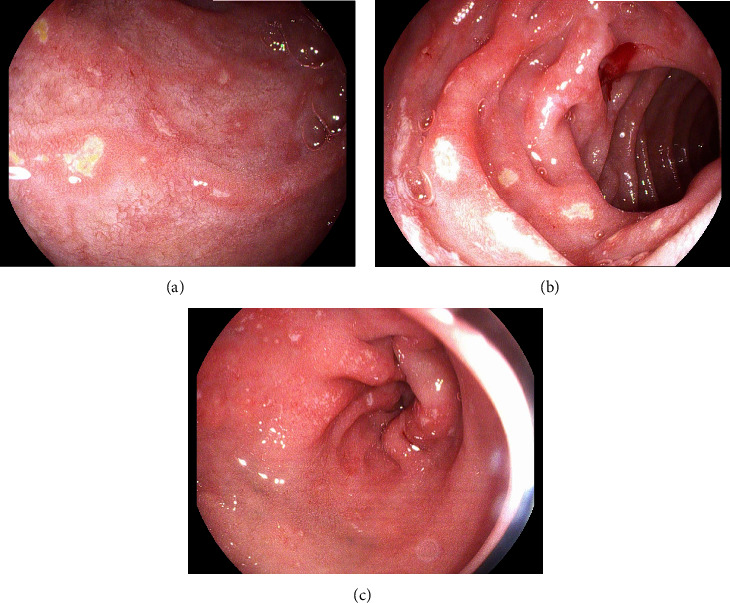
Duodenal bulb, 2^nd^ portion of the duodenum, and gastric antrum with multiple erosions and ulcerations.

**Figure 4:**
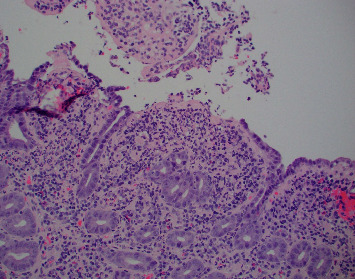
Duodenal mucosa with active erosive chronic duodenitis, villous blunting and surface epithelium replaced by neutrophils consistent with duodenal Crohn's disease, 200x.

Colonoscopy was notable for multiple, nonbleeding, round, and shallow ulcers measuring between 3–7 mm with extensive inflammation and loss of vascularity throughout rectum. Extensive erythema, friability, and shallow ulcerations were noted extending from the anus up to 22 cm, at which point the procedure was aborted due to significant inflammation and the inability to traverse the rectum (Figures 1(a) and 1(b)). Subsequent pathology showed chronic and active colitis with plasma cells and crypts full of neutrophils, consistent with a definitive diagnosis of Crohn's disease (Figure 2). The Crohn's disease activity index (CDAI) score was between 220–450, consistent with moderate-to-severe Crohn's disease. Esophagogastroduodenoscopy showed multiple nonbleeding, round, clean-based, and shallow ulcers ranging between 3–7 mm in the duodenal bulb and the 1^st^ and 2^nd^ parts of the duodenum (Figures 3(a)–3(c)). Pathology showed fragments of duodenal mucosa with focal ulceration and acute inflammation with surface epithelium replaced by neutrophils and plasma cells, as well as moderately chronic, focally active gastritis with negative *H pylori*, establishing a diagnosis of gastroduodenal Crohn's disease (Figure 4). The patient was started on solumedrol and discharged on prednisone, mesalamine enemas, and pantoprazole with follow-up withplans to start infliximab. The patient missed his follow-up and presented 10 days later with diffuse abdominal pain, bilious emesis, and 10–12 episodes of bloody diarrhea daily. White count was 17,900/mm^3^ with ESR 71, CRP 51.59, and calprotectin 3327. CT A/P showed wall thickening and a mild inflammatory fat stranding adjacent to the left colon, now involving the splenic flexure and rectum with increasing inflammatory changes since last admission. The patient was switched to IV methylprednisolone in-house and discharged on prednisone with outpatient adalimumab infusions. This medication switch was made due to an easier home administration regimen, given concerns about noncompliance. He was seen a month later and was doing well with no symptoms. He was then started on azathioprine. His CRP and ESR remained normal, and we suspect his disease is currently under control. The patient was unfortunately lost to follow-up, and subsequent upper and lower endoscopies could not be carried out to evaluate clinical remission.

## 3. Discussion

Symptomatic gastroduodenal CD accounts for approximately less than 4% of all patients affected by CD, with most patients presenting with concurrent large bowel involvement [4]. Our patient presented with CD involving the gastric antrum, the proximal duodenum, and most of the colon with poor response to steroids and mesalamine, returning with an exacerbation. However, subsequent adalimumab and azathioprine therapy resulted in a significantly improved response.

Current literature is bereft of controlled studies assessing the efficacy of medication for the treatment of CD in the upper gastrointestinal tract and, as a result, treatment is based on a combination of distal disease activity and clinical experience [7]. Aside from acid suppression and corticosteroids for flares, emerging cases of biological therapy with antitumor necrosis factor-alpha (TNFα) medications have recently yielded promising results, with both infliximab and adalimumab showing clinical effectiveness in complicated CD patients [8–11]. One key prospective trial showed that, among 19 patients with gastroduodenal CD, 72.7% of those treated with infliximab or adalimumab biological therapy resulted in mucosal healing at 12 weeks post-treatment compared to 12.5% of patients given conventional therapy [12]. Our patient never followed up for infliximab therapy, resulting in his returning with an exacerbation. Our decision to then begin adalimumab therapy was based on our impression that adalimumab would prove a better medication due to easier home administration and its comparable efficacy to infliximab in studies on small bowel CD treatments [13, 14]. This proved successful as he maintained remission for the months to follow. This approach advocates for either infliximab or adalimumab as the initial anti-TNF medication, with clinical judgement and patient factors determining the drug of choice.

It was the concurrent presence of abdominal pain and emesis that prompted an EGD in our patient which then identified findings consistent with gastroduodenal CD. However, as the disease is often asymptomatic in adults, an EGD is not routinely performed in this population to investigate possible IBD pathology. This results in many CD lesions of the upper GI tract being missed and discovered only later in the disease course if symptoms clinically warrant an upper endoscopy by the overseeing gastroenterologist. The European Crohn's and Colitis Organisation (ECCO) consensus recommends investigation of the small bowel in a newly diagnosed distal-CD patient to determine the extent of upper GI involvement [15]. In a prospective study of 119 adults with new or previously known CD, 16% of patients were found to have upper GI CD, with 63% of them being asymptomatic at the time [13]. Sakuraba et al. performed a retrospective study of 138 distal-CD patients who underwent EGD for any upper GI symptoms, with a significant finding of 51.3% of patients having CD-specific lesions of the upper GI tract [16]. This emphasizes the importance of including routine upper endoscopy in the diagnostic evaluation of ileocolonic CD patients in order to investigate the presence, distribution, and severity of gastroduodenal involvement.

It bears mentioning that CD is a difficult condition to treat, with vast financial resources being used to research and develop more efficacious therapies. The current consensus for moderate-to-severe CD is the use of biologics early in the disease course to decrease the risk of steroid usage, hospitalizations, and surgeries and to increase quality of life [17]. The categories of current available biologics for CD include anti-TNF*α* agents, anti-integrins, and anti-interleukin (IL) 12–23. However, emerging and recently approved therapies for CD have yielded significant headway in disease management. Risankizumab is a monoclonal antibody directed against the p19 subunit of IL-23 that has recently been approved by the Food and Drug Administration for moderate-to-severe Crohn's disease. Ustekinumab inhibits both IL-12 and IL-23 and has also been effective in the induction and remission of moderate-to-severe Crohn's disease [18] since 2016. Going further back, vedolizumab is an anti-integrin biologic approved in 2014 for treating moderate-to-severe Crohn's disease. Several other janus kinase (JAK) inhibitor, antitrafficking agents, and sphingosine-1-phosphate receptor modulator therapies are currently in development for the more efficacious treatment of CD [19].

Despite EGD and biopsy being the gold standard for diagnosing gastroduodenal CD, both endoscopy and histopathology may often yield nonspecific results which then may cast doubt to establishing a definitive diagnosis. One therefore needs to be vigilant when suspecting gastroduodenal CD by combining these findings with clinical acumen, as the diagnosis warrants early intervention with biological treatment to promote and/or maintain remission. Endoscopic findings range from edema, enanthem, and longitudinal/irregular/serpiginous erosions to “cobblestoning,” mucosal nodularity, duodenal stenosis/“notching,” and a “bamboo-joint-like” appearance [7, 20, 21]. Affected areas are frequently confined to the antrum, pylorus, and proximal duodenum with the proximal stomach usually spared [22]. Histological findings also vary and range from nonspecific focal inflammatory changes, acute and chronic inflammation, and focal gastritis to lymphoid aggregates, epithelioid granulomas, and duodenitis with or without granulomas. Due to such variability and the focal distribution of disease presentation, biopsies should be performed in both endoscopically normal and abnormal areas. While noncaseating granulomas are nearly pathognomonic for CD, their absence does not rule out gastroduodenal pathology. They are most frequently located in the stomach rather than in the duodenum, with their presence reported between 7%–87.7% and 0–49% in those areas, respectively [21]. Focal gastritis in the absence of *H. pylori* has also been described in several studies as suggestive of gastric CD, located more frequently in the antrum [23, 24]. Other studies further note that even chronic *H. pylori* negative gastritis has been reported at high frequences in CD patients to establish a gastroduodenal disease component [13, 25]. Our patient did not have granulomas identified on EGD. However, his nonspecific biopsy findings of focal gastritis and ulceration, along with macroscopic findings of multiple shallow ulcers on endoscopy, were sufficient to establish the diagnosis of gastroduodenal involvement of CD. We again emphasize the importance of concurrent, early endoscopic evaluation in CD patients to assess for upper GI involvement, as the presence of positive findings warrants the initiation of more aggressive medical and/or surgical interventions. Furthermore, the frequent obscurity in identifying gastroduodenal CD encourages the integration and amalgamation of clinical, endoscopic, histological, and radiological findings along with practitioners' clinical experience and gestalt in order to establish a confirmatory diagnosis of this disease.

## Data Availability

The case report data, including pathology, hospital course, and cited studies used to support the findings of this study are included within the article and are also available from the corresponding author upon request. The personal protected patient information used to support the findings of this study is restricted by the University at Buffalo Institutional Review Board in order to protect patient privacy. Data are available from the primary author clive.miranda91@gmail.com for researchers who meet the criteria for access to confidential data.

## References

[1] Loftus E. V. (2016). Update on the incidence and prevalence of inflammatory bowel disease in the United States. *Gastroenterology and Hepatology*.

[2] Nikolaus S., Schreiber S. (2007). Diagnostics of inflammatory bowel disease. *Gastroenterology*.

[3] Van Assche G., Dignass A., Panes J. (2010). The second European evidence-based consensus on the diagnosis and management of Crohn’s disease: definitions and diagnosis. *Journal of Crohn’s and Colitis*.

[4] Reynolds H. L., Stellato T. A. (2001). Crohn’s disease of the foregut. *Surgical Clinics of North America*.

[5] Ingle S. B., Pujari G. P., Patle Y. G., Nagoba B. S. (2011). An unusual case of Crohn’s disease with isolated gastric involvement. *Journal of Crohn’s and Colitis*.

[6] Gottlieb C., Alpert S. (1937). Regional jejunitis. *American Journal of Roentgenology*.

[7] Pimentel A. M., Rocha R., Santana G. O. (2019). Crohn’s disease of esophagus, stomach and duodenum. *World Journal of Gastrointestinal Pharmacology and Therapeutics*.

[8] Kim Y. L., Park Y. S., Park E. K. (2014). Refractory duodenal Crohn’s disease successfully treated with infliximab. *International Researchers*.

[9] Odashima M., Otaka M., Jin M. (2007). Successful treatment of refractory duodenal Crohn’s disease with infliximab. *Digestive Diseases and Sciences*.

[10] Gaggar S., Scott J., Thompson N. (2012). Pyloric stenosis associated Crohn’s disease responding to adalimumab therapy. *World Journal of Gastrointestinal Pharmacology and Therapeutics*.

[11] Tursi A. (2011). Duodenal Crohn’s disease successfully treated with adalimumab. *Endoscopy*.

[12] Annunziata M. L., Caviglia R., Papparella L. G., Cicala M. (2012). Upper gastrointestinal involvement of Crohn’s disease: a prospective study on the role of upper endoscopy in the diagnostic work-up. *Digestive Diseases and Sciences*.

[13] Bouhnik Y., Carbonnel F., Laharie D. (2018). Efficacy of adalimumab in patients with Crohn’s disease and symptomatic small bowel stricture: a multicentre, prospective, observational cohort (CREOLE) study. *Gut*.

[14] Doecke J. D., Hartnell F., Bampton P. (2017). Infliximab vs. adalimumab in Crohn’s disease: results from 327 patients in an Australian and New Zealand observational cohort study. *Alimentary Pharmacology & Therapeutics*.

[15] Gomollón F., Dignass A., Annese V. (2017). 3rd European evidence-based consensus on the diagnosis and management of Crohn’s disease 2016: Part 1: diagnosis and medical management. *Journal of Crohn’s and Colitis*.

[16] Sakuraba A., Iwao Y., Matsuoka K. (2014). Endoscopic and pathologic changes of the upper gastrointestinal tract in Crohn’s disease. *BioMed Research International*.

[17] Klenske E., Bojarski C., Waldner M., Rath T., Neurath M. F., Atreya R. (2019). Targeting mucosal healing in Crohn’s disease: what the clinician needs to know. *Therap Adv Gastroenterol*.

[18] Feagan B. G., Sandborn W. J., Gasink C. (2016). Ustekinumab as induction and maintenance therapy for Crohn’s disease. *New England Journal of Medicine*.

[19] Al-Bawardy B., Shivashankar R., Proctor D. D. (2021). Novel and emerging therapies for inflammatory bowel disease. *Frontiers in Pharmacology*.

[20] Ingle S. B., Adgaonkar B. D., Jamadar N. P., Siddiqui S., Hinge C. R. (2015). Crohn’s disease with gastroduodenal involvement: diagnostic approach. *World Journal of Clinical Cases*.

[21] Nomura Y., Moriichi K., Fujiya M., Okumura T. (2017). The endoscopic findings of the upper gastrointestinal tract in patients with Crohn’s disease. *Clin J Gastroenterol*.

[22] Kefalas C. H. (2003). Gastroduodenal Crohn’s disease. *Baylor University Medical Center SAVE Proceedings*.

[23] Magalhães-Costa M. H. D., Reis B. R. D., Chagas V. L. A., Nunes T., Souza H. S. P., Zaltman C. (2014). Focal enhanced gastritis and macrophage microaggregates in the gastric mucosa: potential role in the differential diagnosis between Crohn’s disease and ulcerative colitis. *Arquivos de Gastroenterologia*.

[24] Oberhuber G., Püspök A., Oesterreicher C. (1997). Focally enhanced gastritis: a frequent type of gastritis in patients with Crohn’s disease. *Gastroenterology*.

[25] Sonnenberg A., Genta R. M. (2012). Low prevalence of Helicobacter pylori infection among patients with inflammatory bowel disease. *Alimentary Pharmacology & Therapeutics*.

